# Interstitial Pneumonia With Autoimmune Features (IPAF)

**DOI:** 10.3389/fmed.2019.00209

**Published:** 2019-09-27

**Authors:** Ligia Fernandes, Mouhamad Nasser, Kais Ahmad, Vincent Cottin

**Affiliations:** ^1^Departamento do Tórax, Centro Hospitalar Lisboa Norte, Lisbon, Portugal; ^2^Department of Respiratory Medicine, National Reference Center for Rare Pulmonary Diseases, Hospices Civils de Lyon, Lyon, France; ^3^Claude Bernard Lyon 1 University, University of Lyon, INRA, UMR754, Lyon, France

**Keywords:** pulmonary fibrosis, connective tissue disease, classification, autoimmunity, antibody

## Abstract

A significant proportion of patients with interstitial lung disease (ILD) manifest autoimmune features, but do not fulfill the diagnostic criteria for a definite connective tissue disease (CTD). In 2015, the European Respiratory Society (ERS) and American Thoracic Society (ATS) “Task Force on undifferentiated Forms of connective tissue disease-associated interstitial lung disease” proposed classification criteria for a so-called research category of Interstitial Pneumonia with Autoimmune Features (IPAF). These classification criteria were based on a combination of features from three domains: a clinical domain consisting of extra-thoracic features; a serologic domain with specific autoantibodies; and a morphologic domain with imaging patterns, histopathological findings or multi-compartment involvement. Patients meeting IPAF criteria tend to have a history of smoking similar to patients with idiopathic pulmonary fibrosis. The most frequent clinical and serological markers of autoimmune features are Raynaud' phenomenon and positive antinuclear antibodies, respectively. Non-specific interstitial pneumonia is the predominant radiologic and histopathologic pattern, although patients meeting IPAF criteria through the clinical and serologic domains may also have a usual interstitial pneumonia pattern. Management should be carefully individualized on a case-by-case basis in keeping with the wide heterogeneity of IPAF and lack of evidence in this particular subgroup of patients. Prognosis is generally intermediate between that of idiopathic pulmonary fibrosis and connective tissue disease-associated interstitial lung disease, but substantially variable according to the predominant histologic and radiologic patterns. As acknowledged by the Task Force, the proposed classification scheme of IPAF is a research concept that will need revision and refinement based on data to better inform prognostication and patient care.

A sizable proportion of patients with interstitial lung disease (ILD) presents with clinical, serological, and/or radiological features suggestive of connective tissue disease (CTD), but lack features to meet the established diagnostic criteria of defined CTDs (1, 2). In 2015, the European Respiratory Society (ERS) and American Thoracic Society (ATS) “Task Force on undifferentiated Forms of connective tissue disease-associated interstitial lung disease” proposed classification criteria for a so-called research category of Interstitial Pneumonia with Autoimmune Features (IPAF) (3). The classification of IPAF can therefore be considered an overlap between idiopathic interstitial pneumonia especially idiopathic pulmonary fibrosis (IPF) and CTD-ILDs (Figure 1).

**Figure 1:**
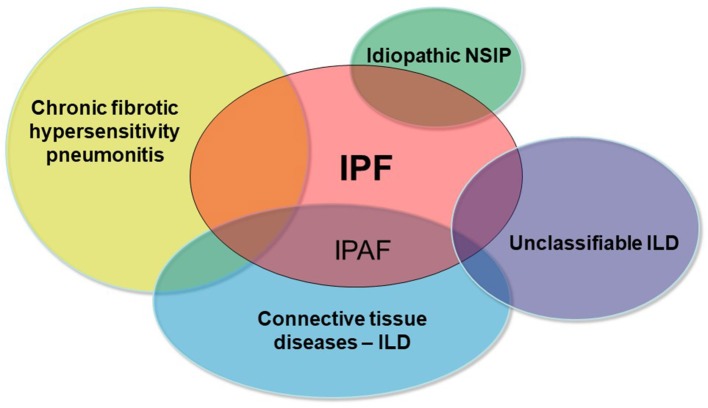
Schematic representation of the main differential diagnoses of pulmonary fibrosis. IPAF represents the overlap between IPF and CTD-ILDs.

## Epidemiology

It has been estimated that up to 25% of patients with features of a systemic autoimmune disease do not fulfill the American College of Rheumatology (ACR) classification criteria for CTD (4).

On the other hand, in the absence of a defined CTD, 10–20% of patients with idiopathic interstitial pneumonia have systemic symptoms and serologic abnormalities suggestive of an autoimmune process. Therefore, worldwide experts from different medical specialities have conceptualized this entity as an undifferentiated CTD-associated ILD, lung-dominant CTD, and autoimmune-featured ILD, using different but overlapping criteria and terminology (1, 5–9).

In 2015, a European Respiratory Society (ERS)/American Thoracic Society (ATS) “Task Force on Undifferentiated Forms of Connective Tissue Disease-associated ILD” proposed a common nomenclature and criteria to describe these patients (3). The concept of idiopathic pneumonia with autoimmune features (IPAF) was thus coined. These proposed criteria created a research platform for standardization of ILD patients harboring autoimmune features that allows further epidemiological studies and better understanding of its prognostic, therapeutic, and pathophysiologic implications (10–12).

The prevalence of IPAF varies between 7 and 34% of all ILDs depending mainly on the population studied and the patient recruitment profile (10–12).

With regards to demographic characteristics, the mean age varies from 60 to 65 years, with balanced gender, although some studies reported a younger mean age (~55 years) and a predominance of white non-smoking women (10–12). These characteristics differ from those observed in CTD-ILD, where patients are predominantly females and younger; and from patients with IPF, who tend to be predominantly males and older. Moreover, IPAF patients are more frequently smokers or ex-smokers, unlike patients with UCTD-ILD, likely related to the greater percentage (~30%) of cases with a usual interstitial pneumonia (UIP) pattern that meet IPAF criteria (5, 12).

## Definition and Diagnosis

### Previous Terminologies

Prior to the consensus criteria for IPAF, there were four proposed terminologies used to define related entities, as showed in Table 1, describing an overlapping although not identical population (13).

**Table 1:** Previous proposed diagnostic criteria for undifferentiated CTD-associated ILD and similar conditions.

**Concept**	**References**	**Diagnostic criteria**	**Main findings**
Undifferentiated connective tissue disease associated-ILD, broader definition	Kinder et al. (5)	**Symptoms associated with CTD** At least one of: (1) Raynaud's phenomenon; (2) arthralgias/multiple joint swelling; (3) photosensitivity; (4) unintentional weight loss; (5) morning stiffness; (6) dry mouth or dry eyes (Sicca features); (7) dysphagia; (8) recurrent unexplained fever; (9) gastro-esophageal reflux; (10) skin changes (rash); (11) oral ulceration; (12) nonandrogenic alopecia; (13) proximal muscle weakness; **Positive autoimmune serology** Positive finding of at least one of:(1) ANA; (2) RF; (3) anti-Scl70 antibody; (4) SS-A or SS-B; (5) Jo-1 antibody; (6) ESR (2 times normal), CRP	1. Hypothesis of idiopathic NSIP as a lung manifestation of a UCTD; 2. The majority (88%) of patients previously classified as having idiopathic NSIP had clinical, serologic, radiographic, and pathologic characteristics met the criteria for UCTD.
Undifferentiated connective tissue disease—strict definition	Corte et al. (7)	**Symptoms associated with CTD** At least one of: (1) Raynaud's phenomenon; (2) arthralgias/multiple joint swelling; (3) morning stiffness; (4) dry mouth or dry eyes (Sicca features); (5) proximal muscle weakness **Positive autoimmune serology** Positive finding of at least one of:(1) ANA (high titer); (2) RF (high titer); (3) positive ENA; (4) anti-Scl70 antibody; (5) anti-RNP antibody; (6) anticentromere antibody; (7) SS-A or SS-B; (8) Jo-1 antibody	1. CTD features were not uncommon in IP patients; 2. Less specific diagnostic criteria for UCTD were not useful and associated with a erroneous high prevalence; 3. UCTD diagnosis of was correlated with NSIP histology, without sensitivity or specificity for NSIP, nor association with a survival advantage.
Lung dominant-connective tissue disease	Fischer et al. (9)	Four criteria: 1. NSIP, UIP, LIP, OP, and DAD (or DIP if no smoking history), by surgical lung biopsy specimen or suggested by high-resolution CT; 2. Insufficient extrathoracic features of a definite CTD to allow a specific CTD designation; 3. No identifiable alternative etiology; 4. Any one of the following autoantibodies or at least two of the histopathology features: **Autoantibodies** High-titer ANA (>1:320) or RF (>60 IU/mL), Nucleolar-ANA, Anti-CCP, Anti-Scl-70, Anti-Ro, Anti-La, Anti-dsDNA, Anti-Smith, Anti-RNP, Anti-tRNA synthetase (e.g., Jo-1, PL-7, PL-12, and others), Anti-PM-Scl, anticentromere **Histopathology features** Lymphoid aggregates with germinal centers, extensive pleuritis, prominent plasmocytic infiltration, and dense perivascular collagen	Advantages of these criteria: - Objective and measurable; - Nonspecific symptoms, nonspecific inflammatory markers, and low-titer ANA or RF were not included due to its common occurrence in patients without definite CTD; - The term “lung-dominant CTD” was distinct from the idiopathic group of IP and acknowledged a new entity manifested by systemic autoimmunity that could not be designated as a definable CTD; - The diagnosis of lung-dominant CTD provided a framework for research regarding natural history, pathobiology, treatment, and prognosis.
Autoimmune-featured interstitial lung disease (AIF-ILD)	Vij et al. (6)	**Symptoms (one or more of the following)** Dry eyes/dry mouth; gastroesophageal reflux; weight loss; leg/foot swelling; joint pain/swelling; rash photosensitivity; dysphagia; hand ulcers; mouth ulcers; Raynaud phenomenon; morning stiffness; proximal muscle weakness; **Serologic test (one or more positive result of the following)** Antinuclear antibody titer 1:160; rheumatoid factor; aldolase; Anti-Ro antibody; Anti-La antibody; Anti-neutrophil cytoplasmic antibody; Creatine kinase; Anti-double-stranded DNA; Anti-Scl-70; Anti-ribonucleoprotein antibody; Anti-Smith antibody; Anti-cyclic citrullinated peptide antibody; Anti-Jo-1 antibody.	- Demographic profile for gender and age of AIF-ILD group shared similarities with IPF group, but was different from CTD-ILD group; - The most frequent radiological finding in AIF-ILD patients was UIP (62%).

*(U)CTD, (undifferentiated) connective tissue disease; ANA, antinuclear antibody; RF, rheumatoid factor; ENA, extractable nuclear antigen; RNP, ribonucleoprotein; SS-A, anti-Ro; SS-B, anti- La; ESR, erythrocyte sedimentation rate; CRP, C-reactive protein, IP, interstitial pneumonia, NSIP, non-specific interstitial pneumonia; UIP, usual interstitial pneumonia; LIP, lymphocytic interstitial pneumonia; OP, organizing pneumonia; DAD, diffuse alveolar damage; ILD, interstitial lung disease; CTD-ILD, connective tissue disease associated-ILD; IPF, idiopathic pulmonary fibrosis*.

### IPAF Classification

The current consensus definition of IPAF proposed by Fischer et al. in 2015 includes three criteria (3):

1. Radiological or histopathological evidence of interstitial pneumonia *and*,
2. Complete clinical evaluation excluding other etiologies for interstitial pneumonia *and*,
3. Incomplete features of a defined CTD.

To meet criteria for IPAF, cases must fulfill the three *a priori* requirements, in addition to a minimum of *one feature from at least two* of the following domains (Table 2):

- A. Clinical domain (specific clinical features);
- B. Serologic domain (specific circulating autoantibodies);
- C. Morphologic domain (specific chest imaging features, histopathological features, or multi-compartment involvement).

**Figure 2:**
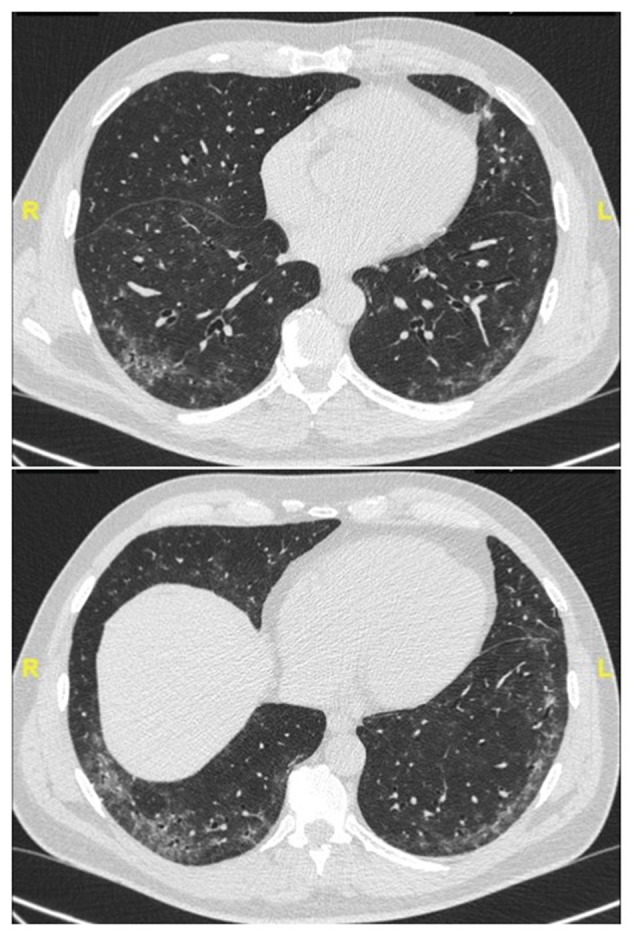
Chest CT (lung window) in a 59-year old, non-smoker male patient with recent onset of Raynaud' phenomenon and non-specific interstitial pneumonia at lung biopsy (biopsy courtesy of Prof F. Thivolet-Béjui, Lyon) fulfilling IPAF criteria. No overt CTD has developed after a follow-up of 2 years.

**Figure 3:**
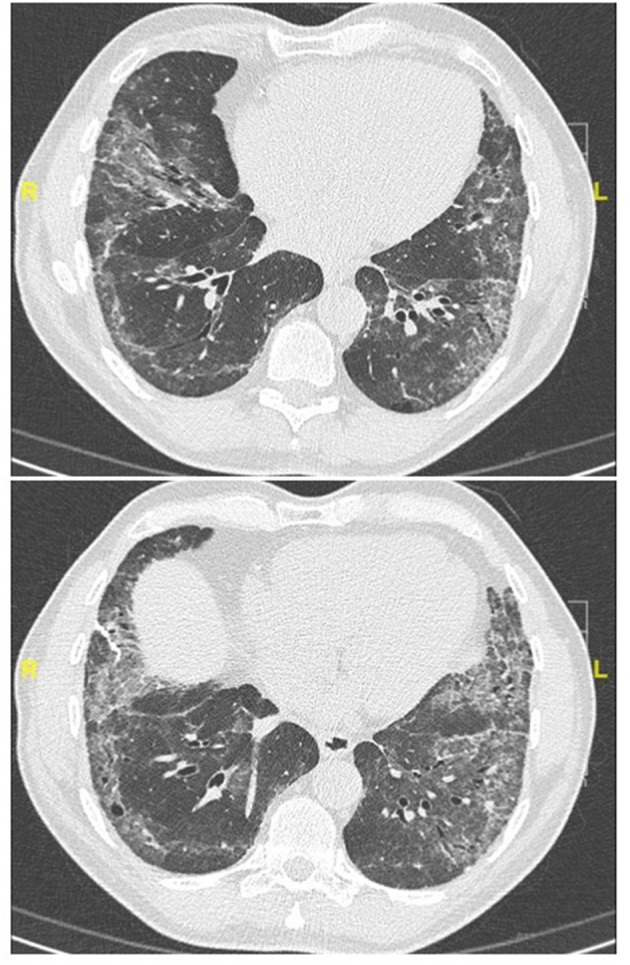
Chest CT (lung window) in a 62-year old, non-smoker male patient fulfilling IPAF criteria, with puffy fingers, mechanics' hands, gastro-esophageal reflux, high titer (1:1280) antinuclear antibodies. Lung biopsy demonstrated a pattern of probable usual interstitial pneumonia without fibroblastic foci and a paucity of lymphocytic inflammation (biopsy courtesy of Prof F. Thivolet-Béjui, Lyon).

**Figure 4:**
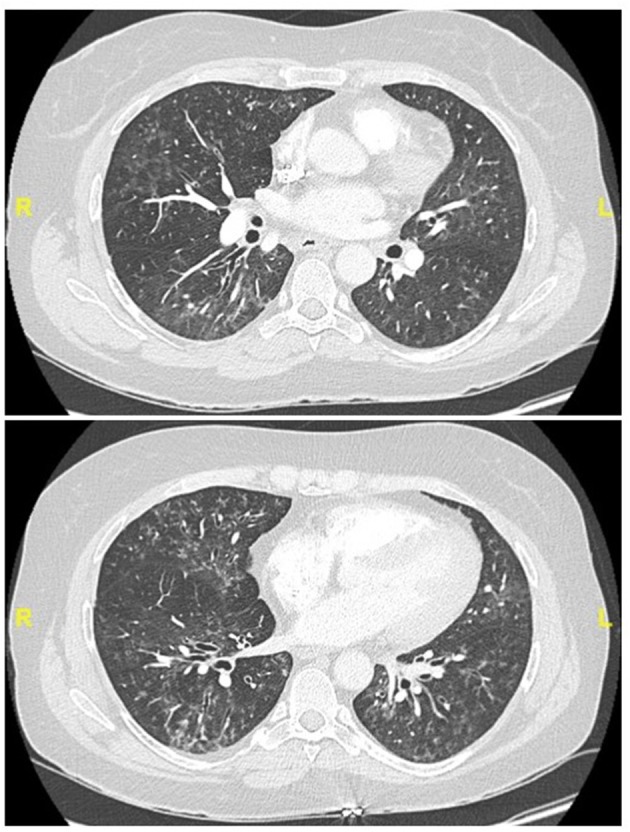
Chest CT (lung window) in a 55-year old, non-smoker female patient fulfilling IPAF criteria, with Raynaud' phenomenon (clinical domain) and a complex pattern of non-specific interstitial pneumonia and lymphocytic bronchiolitis at biopsy (multicompartment involvement, morphologic domain).

Representative examples are provided in Figures 2–4.

**Table 2:** Classification criteria for interstitial pneumonia with autoimmune features [adapted from Fischer et al. (3)].

**A. Clinical domain**	**B. Serologic domain**	**C. Morphologic domain**
1. Distal digital fissuring (mechanic hands) 2. Distal digital tip ulceration 3. Inflammatory arthritis or polyarticular morning joint stiffness >60 min 4. Palmar telangiectasia 5. Raynaud's phenomenon 6. Unexplained digital oedema 7. Unexplained fixed rash on the digital extensor surfaces (Gottron's sign)	1. ANA ≥1: 320 titer, diffuse, speckled, homogeneous patterns or a) ANA nucleolar pattern (any titer) or b) ANA centromere pattern (any titer) 2. Rheumatoid factor ≥2× upper limit of normal 3. Anti-CCP 4. Anti-dsDNA 5. Anti-Ro (SS-A) 6. Anti-La (SS-B) 7. Anti-ribonucleoprotein 8. Anti-Smith 9. Anti-topoisomerase (Scl-70) 10. Anti-tRNA synthetase (e.g., Jo-1, PL-7, PL-12; others are: EJ, OJ, KS, Zo, tRS) 11. Anti-PM-Scl 12. Anti-MDA-5	1. Suggestive radiology patterns by high-resolution computed tomography (HRCT): a) NSIP b) OP c) NSIP with OP overlap d) LIP 2. Histopathology patterns or features by surgical lung biopsy: a) NSIP b) OP c) NSIP with OP overlap d) LIP e) Interstitial lymphoid aggregates with germinal centers f) Diffuse lymphoplasmacytic infiltration (with or without lymphoid follicles) 3. Multi-compartment involvement (in addition to interstitial pneumonia): a) Unexplained pleural effusion or thickening b) Unexplained pericardial effusion or thickening c) Unexplained intrinsic airways disease d) Unexplained pulmonary vasculopathy

*ANA, antinuclear antibody; HRCT, high-resolution computed tomography; IPAF, interstitial pneumonia with autoimmune features; LIP, lymphoid interstitial pneumonia; NSIP, non-specific interstitial pneumonia; OP, organizing pneumonia; PFT, pulmonary function testing*.

### Signs and Symptoms—What Are Generally the Signs and Symptoms?

According to three published cohorts (10–12), there was a significantly high prevalence of patients meeting clinical and serological criteria. Between 47 and 63% of IPAF patients had at least one clinical sign. The most common clinical signs were Raynaud' phenomenon (28–39%), followed by mechanics' hands (4–29%), arthritis or morning stiffness (16–23%), and Gottron' sign (5–18%) (14).

### Serology

More than 90% of patients with IPAF have at least one of the serological criteria (10). The main auto-antibody found in several series was positive high-titer antinuclear antibodies (48–82%). Anti-SSa, antisynthetase antibodies, as well as high titer rheumatoid factor were also frequent (10–12).

### Imaging and Pathology

The non-specific interstitial pneumonia (NSIP) pattern at HRCT and/or histopathology (41 and 25%, respectively) was the most frequent finding in several series (10, 14–17). However, Oldham et al. found a higher prevalence of the UIP pattern (55% at HRCT; 74% on surgical lung biopsy), an observation that was due to the patient recruitment profile of these centers (12).

Noteworthy, the histological UIP pattern observed in IPAF patients has been reported as non-typical, with diffuse lymphoplasmacytic infiltration, interstitial lymphoid aggregates, or histological involvement of the airways (10, 18). These inflammatory findings were formerly considered as characteristic histological features for lung-dominant CTD (8), and were based on observed pathological differences between idiopathic UIP and UIP in the setting of CTD (19).

Interestingly, patients meeting IPAF criteria can retrospectively be identified within each category of the interstitial pneumonias heretofore considered idiopathic. In the series by Oldham et al. (12), patients initially characterized as IPF, cryptogenic organizing pneumonia/NSIP and unclassifiable ILD met IPAF criteria (serologic and morphological domains) in 88, 78, and 50% of cases, respectively. Those previously characterized as UCTD-ILD fulfilled all three domains of IPAF criteria in nearly 50% of cases (12).

## Pathophysiology

The pathophysiology of IPAF remains elusive, as no specific studies have been conducted, and it is assumed that pathways involved in IPF and/or in CTD-ILD would be involved in IPAF. It is generally considered that pathophysiologic studies are difficult to design in the absence of clear diagnostic boundaries, and especially in the absence of consensus regarding IPAF being an entity. However, it may be argued that identifying pathways specifically involved in IPAF may in fact contribute to identify IPAF as an entity.

In a study by Newton et al. (20), differences were found between patients with IPAF and those with IPF or CTD-ILD with regard to leukocyte telomere length, MUC5B polymorphism but not TOLLIP polymorphism. Both telomere length and MUC5B polymorphism were associated with survival. Fewer patients with IPAF and CTD-ILD had short telomeres as compared to IPF, but short telomere length in IPAF was associated with faster decline in lung function and lung transplantation, similar to IPF (20). Although it is difficult at this stage to fully understand the significance of these observations, and it is not known whether genetic markers may help guiding treatment indications in the future, these results point to genetic differences between IPAF, IPF, and CTD-ILD.

## Prognosis

Prior to the international IPAF research statement, it was shown that patients with interstitial pneumonia with features of autoimmunity tend to have an improved survival as compared to those without these features, even though only the *Corte* criteria independently predicted improved survival (13).

Survival studies of cohorts of patients meeting the consensus IPAF criteria have found conflicting results. The University of Chicago pulmonary cohort found that patients classified as IPAF had shorter survival than CTD-ILD patients, but a slightly better outcome than patients with IPF (12). When patients were stratified according to the high-resolution computed tomography (HRCT) pattern, patients with non-UIP IPAF pattern had a very similar prognosis to those with CTD-ILD, while disease progression of UIP-IPAF patients resembled that of patients with IPF. The GAP index, a score developed in IPF and based on gender, age, and lung physiology (forced vital capacity (FVC) and diffusing capacity of the lung for carbon monoxide) predicted mortality (12). While the presence of a clinical domain was associated with a decreased mortality risk, the serological and the morphological domains were not associated with a significant increase in mortality risk. Nevertheless, the presence of a multi-compartment feature was a strong predictor of poor outcome.

Conversely, our cohort from the Claude Bernard Lyon University, France, found no significant difference in overall survival between IPAF and IPF patients (10). Amongst patients with IPAF, UIP, or non-specific interstitial pneumonia (NSIP) pattern had no significant impact on survival, while history of smoking was the only factor significantly associated with increased mortality (10). In the cohort from the University of Colorado Rheumatology Clinic, patients experienced no significant decline in FVC or death during the follow-up period. This finding might be attributable to favorable prognostic factors among patients recruited in the study, such as the majority of patients being never-smokers, females, and responsive to effective immunosuppressive therapy (11). In other words, it appears that cohorts from pulmonology departments may be enriched in cases of IPAF with characteristics and outcome close to those of IPF (10, 12), whereas cohorts from rheumatology departments (11) may have characteristics closer to those of CTD-ILD.

In another study, it was observed that a radiological NSIP pattern and a higher age were associated with a poor prognosis compared to other patients classified as IPAF patients with organizing pneumonia or NSIP/organizing pneumonia overlap (16). The radiological-pathological pattern was more predictive of the prognosis than highly specific autoantibodies related to known CTDs (16).

A recent study from South Korea recently confirmed that patients classified as IPAF had a 1-, 3-, and 5-year survival lower than that of CTD-ILD, and better than that of patients with IPF (with fewer acute exacerbations of fibrosis) (21). However, no significant difference in survival was found between patients with IPAF patients and a UIP pattern and those with IPF patients (21), as previously observed in another cohort (12).

As a result of these dissimilarities, longitudinal research using ILD clusters analysis has been performed to identify clinical phenotypes and to predict outcomes. Phenotypic clusters were able to anticipate lung function deterioration and survival, independently of the primary ILD classification (22). IPAF were mostly found in two clusters with a heterogeneous clinical presentation—the cluster of “younger African-American females with elevated antinuclear antibody titres” and in the cluster of “elderly Caucasian male smokers, with severe honeycombing” (22).

In a recent study (17), the presence of a UIP pattern at high resolution computed tomography and/or histopathology was associated with a poor outcome as compared to a non-UIP pattern among patients with IPAF, although in general the diagnosis of IPAF was associated with a better outcome than IPF. Similarly, Yoshimura et al. (23) found that patients with a pattern of NSIP who met criteria for IPAF had a better outcome than those with idiopathic NSIP; patients with UIP and IPAF also had a better outcome than those with IPF (idiopathic UIP—no IPAF).

Dai et al. compared the outcome of patients classified as IPAF to those with idiopathic interstitial pneumonia other than IPF (15). They found that patients with IPAF had worse prognosis as compared to those with non-IPF idiopathic interstitial pneumonia and a better survival than those with IPF. Interestingly, NSIP was the predominant HRCT pattern among patients with IPAF (61%) (15), as in other series (14, 17). In multivariate analysis, several factors including age, smoking history, a pattern of organizing pneumonia at CT, and anti-RNP positivity were independently associated with a worse survival (15).

It has to be emphasized that most of the prognosis studies are limited by the retrospective design. In the only published prospective cohort, IPAF patients had less severe disease at diagnosis and were more frequently women (62%) as compared to those with IPF, however no survival analysis was available (14).

Overall, these finding suggest that the presence of IPAF criteria is associated with a generally better outcome as compared to IPF. Among patients classified as IPAF, the UIP pattern at imaging or histopathology may be associated with a more severe outcome as compared to other patterns especially that of NSIP. Despite this general trend reproduced in a number of cohorts, some significant discrepancy exists between the published series, which may be related to the methodology used to identify CTD features and the referral pattern of the centers. The potential prognostic significance of individual IPAF criteria could not be assessed in the available studies.

## Management

Data regarding IPAF treatment are only limited to case series, and further research is needed to determine the optimal treatment strategy in the IPAF population. As in other ILDs (24), pulmonary rehabilitation, long-term oxygen supplementation therapy if appropriate, and treatment of gastro-esophageal reflux if present are indicated, as well as prevention of infection and bone health assessment.

As IPAF is a research consensus statement and not a disease or a well-defined entity, it is unsure whether a specific management distinct from that of IPF is needed, however research is required to address this question. There have been no randomized controlled trials supporting immunomodulation in IPAF, and the proposed treatment strategies are extrapolated from CTD-ILD studies (25, 26). In one study of patients with unclassifiable ILD, intravenous pulse cyclophosphamide was suggested to stabilize lung function (27); a subset of patients in this study had IPAF and these seemed to benefit more from the treatment regimen, although none of them had a UIP pattern. This suggest that patients with IPAF and a non-UIP pattern may benefit from immunomodulation, however this needs confirmation.

More generally in CTD-ILD, corticosteroids and immunosuppressive agents are considered as the mainstay of treatment (28). Azathioprine and mycophenolate mofetil are associated with improvement or stabilization of lung function with good tolerance (29–31). Similarly, mycophenolate seemed to improve the slope of FVC and of carbon monoxide diffusion capacity in a small cohort of IPAF (32). Calcineurin inhibitors (ciclosporine and tacrolimus) have also been used in combination with corticosteroids (33). Cyclophosphamide is considered as the mainstay of therapy for severe or life-threatening forms of CTD-ILD while rituximab (anti-B-cell CD20 monoclonal antibody) is used as salvage therapy in patients with refractory CTD-ILD (26).

Nintedanib, a tyrosine kinase inhibitor with antifibrotic properties (34), has been approved in IPF, and was recently demonstrated to slow down disease progression in systemic sclerosis-associated ILD (35); approximately half the patients were also receiving mycophenolate in this study. Pirfenidone, another antifibrotic drug approved in IPF (36), is being evaluated in several CTD-ILDs especially systemic sclerosis-associated ILD (https://clinicaltrials.gov identifier: NCT03221257). Both antifibrotic drugs are also currently evaluated in unclassifiable progressive ILD (including IPAF) and fibrosing ILDs with a progressive phenotype despite standard therapy (37–39). As antifibrotic drugs are beneficial in IPF and may be beneficial in CTD-ILD, it is conceivable that a treatment benefit may also be found in subjects with IPAF, however results of the trials are eagerly awaited.

**Figure 5:**
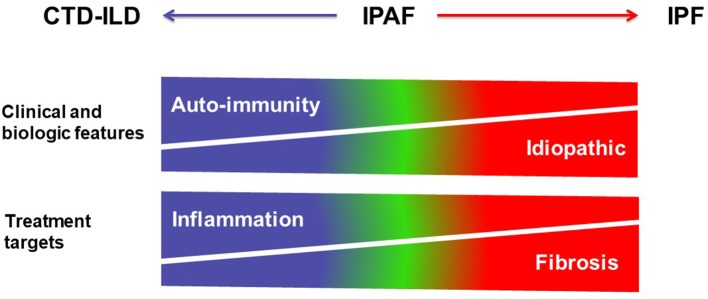
Schematic representation of the spectrum of interstitial lung diseases with or without autoimmunity. CTD-ILDs are characterized by clinical and biologic features of auto-immunity, and mostly treated using corticosteroids and immunosuppressive therapy targeting inflammation, whereas IPF is an idiopathic disease treated using antifibrotic drugs. IPAF is represented within the spectrum between CTD-ILD and IPF in this over-simplistic representation.

Currently, treatment decisions in patients classified as IPAF must be based on careful evaluation of benefit: risk ratio in the individual subject and should ideally be discussed in multidisciplinary setting. The demographic, clinical and autoimmune features, as well as the imaging phenotype should be taken into consideration. Eventually, the choice of first-line therapy may be based on the global assessment of the patient (Figure 5). As an example, cases with a predominantly fibrosing phenotype and with progressive disease might not benefit from corticosteroids and/or immunosuppressive drugs, as their clinical behavior is more comparable to those with IPF; however such treatment may be tried in isolated cases, especially in subjects with a non-UIP pattern, for example when there are individual data to suggest an important component of inflammation based on BAL or histology. The clinician should be reminded however of the general detrimental effect of corticosteroid and/or immunosuppressive therapy in patients with IPF, and not prescribe such treatment in those subjects in whom a working diagnosis of IPF is made. Conversely, although the role of antifibrotic drugs has not yet been specifically studied in the setting of IPAF, patients with a diagnosis of IPF who further fulfill criteria for the IPAF classification often receive antifibrotic therapy. Whether the combination of immunosuppressive therapy and of antifibrotic drugs (40) may be useful in subjects with IPAF will need to be explored in the future.

## Areas of Uncertainties and Controversies

IPAF has raised considerable interest and has highlighted the need for multidisciplinary discussion in the field of CTD-ILD, especially the input of rheumatologists in the ILD clinic (41), and has spurred the dialogue between medical specialties (25).

The concept of IPAF emphasizes that there can be an overlap between IPF and CTD-ILD. Such overlap between entities is common in medicine, for example between the different CTDs (overlap CTD). Among ILDs, another overlap may exist between IPF and chronic fibrotic hypersensitivity pneumonitis (“IPF with exposure to inhaled antigens”) (42).

**Figure 6:**
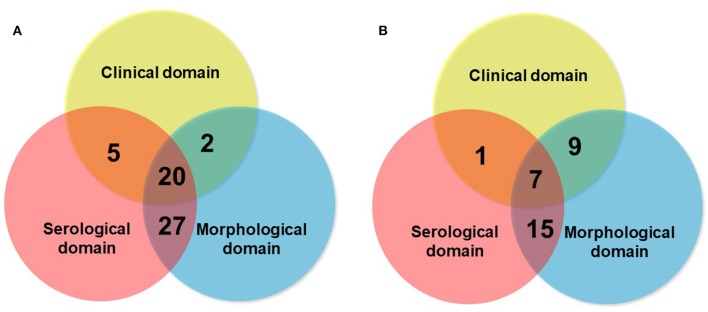
Schematic representation of the respective contribution of the clinical domain (blue circle), serological domain (yellow circle), and morphological domain (blue circle), in two series of IPAF of 54 patients [**A**: Ahmad et al. (10)], and of 32 patients [**B**: Yoshimura et al. (23)].

Although the consensus terminology and definition of IPAF is an important research step in the field, as it provides a uniform classification and criteria for research, a question that remains open is whether IPAF may also represent a clinical diagnosis. The respective contribution of the different domains to the classification as IPAF may vary between series (Figure 6), suggesting variations in referral patterns. Published studies show that the proportion of patients classified as IPAF who progress to a diagnosis of CTD-ILD exist however they represent a minority (10–20%) (16, 43, 44), suggesting that IPAF may be helpful in taking managing decisions in patients presenting with ILD and mild CTD features, therefore addressing a clinical unmet need. Furthermore, many cases of IPAF do not have a pattern of NSIP, and IPAF can therefore not be considered as only a variant of idiopathic NSIP featuring a positive serology.

The strongest argument in favor of IPAF being a potential clinical diagnosis would be the demonstration of a unique outcome (i.e., distinct from that of both IPF and CTD-ILD), which data tend to confirm. As discussed earlier, in the future pathophysiologic considerations and especially genetic studies (20) might also support the concept of IPAF as an entity by demonstrating differences in pathways involved or distinct genetic signatures. Conversely, the heterogeneity of the published series of IPAF suggests that not all patients classified with IPAF may suffer from the same condition. For example, an 80-year-old ex-smoker male with UIP, Raynaud' phenomenon and positive ANA (1/320), and a 40-year-old female, never-smoker, with same findings, likely have different outcomes and management needs. Age and sex are key determinants of diagnosis and prognosis in subjects with ILD and may need to be better taken into account when considering a classification as IPAF. If there is such a thing as a distinct condition of IPAF, then it either may present with various phenotypes, or the diagnostic criteria need to be refined. Subjects with UIP-IPAF have a poor outcome as compared to those with non-UIP IPAF, and those may need to be separated (45).

Many aspects remain controversial, including some of the individual items (45, 46). Current criteria represent a compromise between specificity and sensitivity. Items with very high specificity for CTD may arguably be almost sufficient to consider a diagnosis of CTD [ex: presence of anti-tRNA-synthetase antibodies with mechanics' hands (47)], whereas those with high sensitivity may be criticized for lack of specificity for CTD (ex: presence of rheumatoid factor, or anti-SSB antibodies). Some additional items might be considered [ex: esophageal dysmotility, lymphocytic bronchiolitis on biopsy; proximal muscle weakness with myalgia (48)]. Antineutrophil cytoplasmic antibodies (ANCAs) were excluded from the IPAF classification as they are associated with systemic vasculitis, not CTD, however the combination of ILD and ANCAs and anti-myeloperoxydase specificity (49) shares with IPAF many issues related to the overlap between ILD and a chronic, systemic disease.

The classification further fails to capture some combinations of features that may speak to the clinician while not fulfilling the criteria for IPAF; for example, an asymptomatic 45-year-old man with non-UIP pattern at HRCT and positive anti-CCP antibodies fits the diagnostic criteria of IPAF, whereas another patient with similar clinical and serological manifestations but UIP pattern at HRCT doesn't, despite UIP being the predominant pattern in rheumatoid arthritis-ILD. Clustering in time of the manifestations (ex: concomitant apparition of arthralgia and gastro-esophageal reflux in a patient with new-onset ILD) may also be meaningful clinically yet is not captured in IPAF criteria (Prof A. U. Wells, personal communication).

Due to its lack of specificity, the multi-compartment involvement subdomain is the most problematic among other domains, raising concerns about its relevance as an independent criterion *per se*. For example, it is often difficult to decipher whether the presence of airway disease is in favor of underlying CTD (with what diagnostic modalities? How to manage the confounding effect of tobacco smoking? etc.). Similar comments can be made regarding the item of unexplained vasculopathy, for which more objective criteria would be required. Severely altered gas exchanges contrasting with preserved lung volumes in the absence of emphysema suggests the presence of vasculopathy, and a criterion of FVC/carbon monoxide diffusing capacity ratio >1.6 might be considered.

Some of the items are dependent to a large extent on the effort made to look for CTD features. Nailfold capillaroscopy, biopsy of accessory salivary glands (10), or consultation with a rheumatologist or a dermatologist, often identify CTD features that may alter the eventual diagnosis, yet they cannot be recommended in all patients with ILD. When to stop in the quest of an underlying CTD often impacts the eventual diagnosis (50).

## Conclusion

In conclusion, the research classification of IPAF is an initial step for the uniform and standardized classification of patients with ILD and autoimmune features in the absence of overt CTD. With data from retrospective and prospective studies, the criteria of IPAF may be refined based on accumulated evidence. It is not yet clear how patients who fulfill criteria for IPAF should be treated. The ongoing phase II trial with pirfenidone in unclassifiable ILD (including IPAF) may provide clues as to whether pirfenidone may be beneficial. The definition of IPAF has already shed light on the importance of a thorough evaluation of patients with apparently idiopathic ILD and on the value of the interaction between medical specialties.

## Author Contributions

All four authors meet ICMJE criteria for authorship, (1) substantial contributions to the conception or design of the work, or the acquisition, analysis or interpretation of data for the work, (2) drafting the work or revising it critically for important intellectual content, (3) provide approval for publication of the content, and (4) agree to be accountable for all aspects of the work in ensuring that questions related to the accuracy or integrity of any part of the work are appropriately investigated and resolved.

### Conflict of Interest

The authors declare that the research was conducted in the absence of any commercial or financial relationships that could be construed as a potential conflict of interest.
